# Supplementary material for: Molecular identity of proprioceptor subtypes innervating different muscle groups in mice
Source: Nat Commun. 2022 Nov 11;13:6867. doi: 10.1038/s41467-022-34589-8 (PMC9652284; doi:10.1038/s41467-022-34589-8)
Supplement: Supplementary file 1 — Supplementary Information [file 41467_2022_34589_MOESM1_ESM.pdf]

Supplementary Figure 1

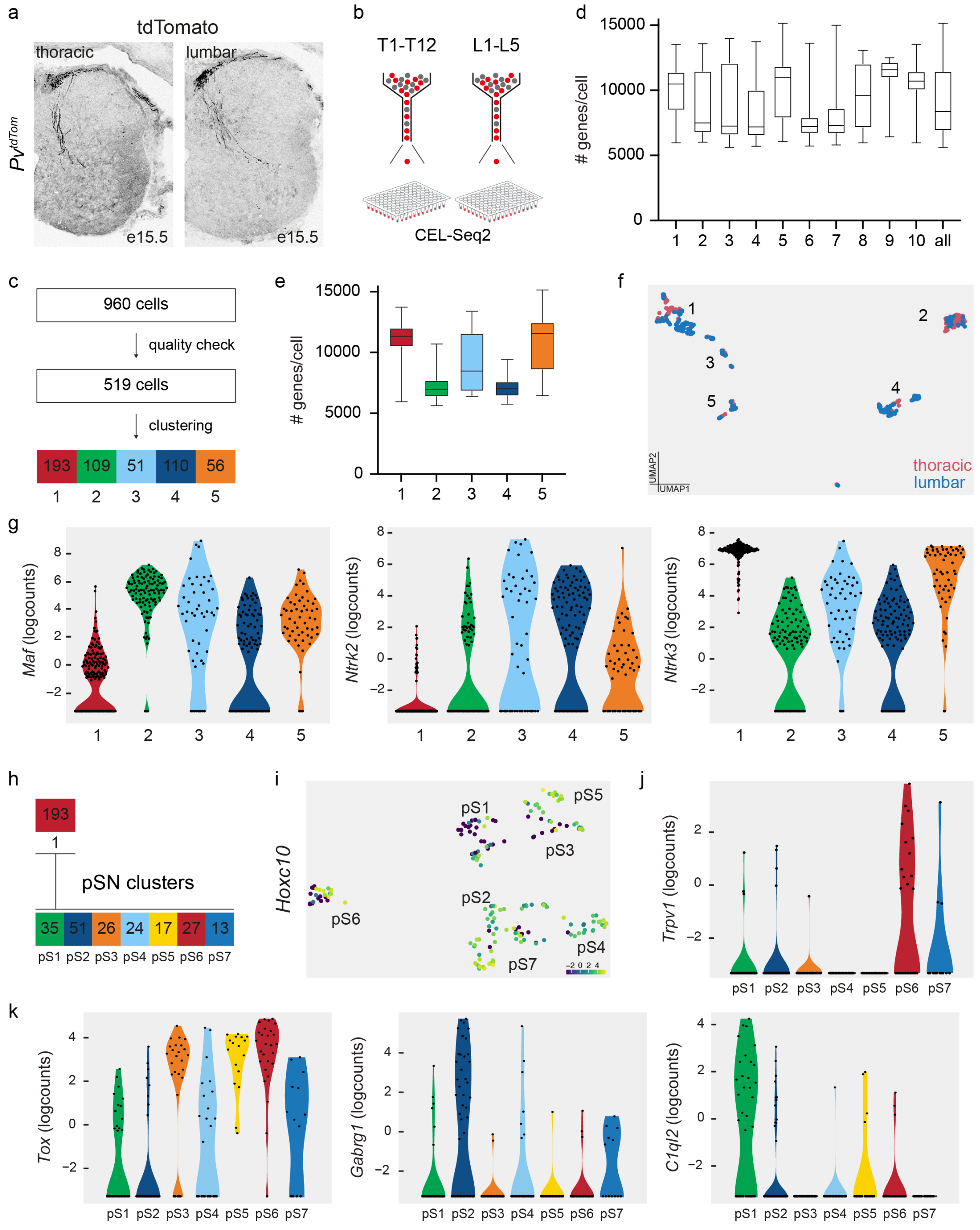

scRNA-seq analysis of e15.5 proprioceptors. a) Representative images of tdTomato^{+} afferents in thoracic and lumbar spinal cord from e15.5 *Pv^{tdTom}* mice. b) Schematic representation of the single cell sorting strategy for sensory neurons dissociated from thoracic and lumbar DRG of e15.5 *Pv^{tdTom}* mice. c) Number of e15.5 *Pv^{tdTom}* DRG neurons sorted, analyzed after quality control and assigned to each cluster after

bioinformatic analysis. d) Boxplots representing the number of genes per cells found in each 96 well plate (#1 to #10) and on average in all plates ("all"). The boxes represent the 1st and 3rd quartiles (bounds of box), median (horizontal line), and whiskers extend to further than 1.5*IQR (interquartile range) from the hinges. e) Boxplots representing the number of genes per cells found in each cluster. The boxes represent the 1st and 3rd quartiles (bounds of box), median (horizontal line), and whiskers extend to further than 1.5*IQR (interquartile range) from the hinges. f) UMAP visualization of proprioceptor clusters color coded according to thoracic (red) and lumbar (blue) origin of the cells. g) Violin plots showing expression (logcounts) of *Maf*, *Ntrk2,* and *Ntrk3* in clusters 1-5. h) Number of C1 neurons re-clustered and assigned to proprioceptive clusters (pS) 1-7. i) UMAP visualization of *Hoxc10* expression (logcounts) in proprioceptor clusters. j) Violin plots showing expression (logcounts) of *Trpv1* in proprioceptor clusters pS1-pS7. k) Violin plots showing expression (logcounts) of *Tox, Gabrg1,* and *C1ql2* in proprioceptor clusters pS1-pS7.

Supplementary Figure 2

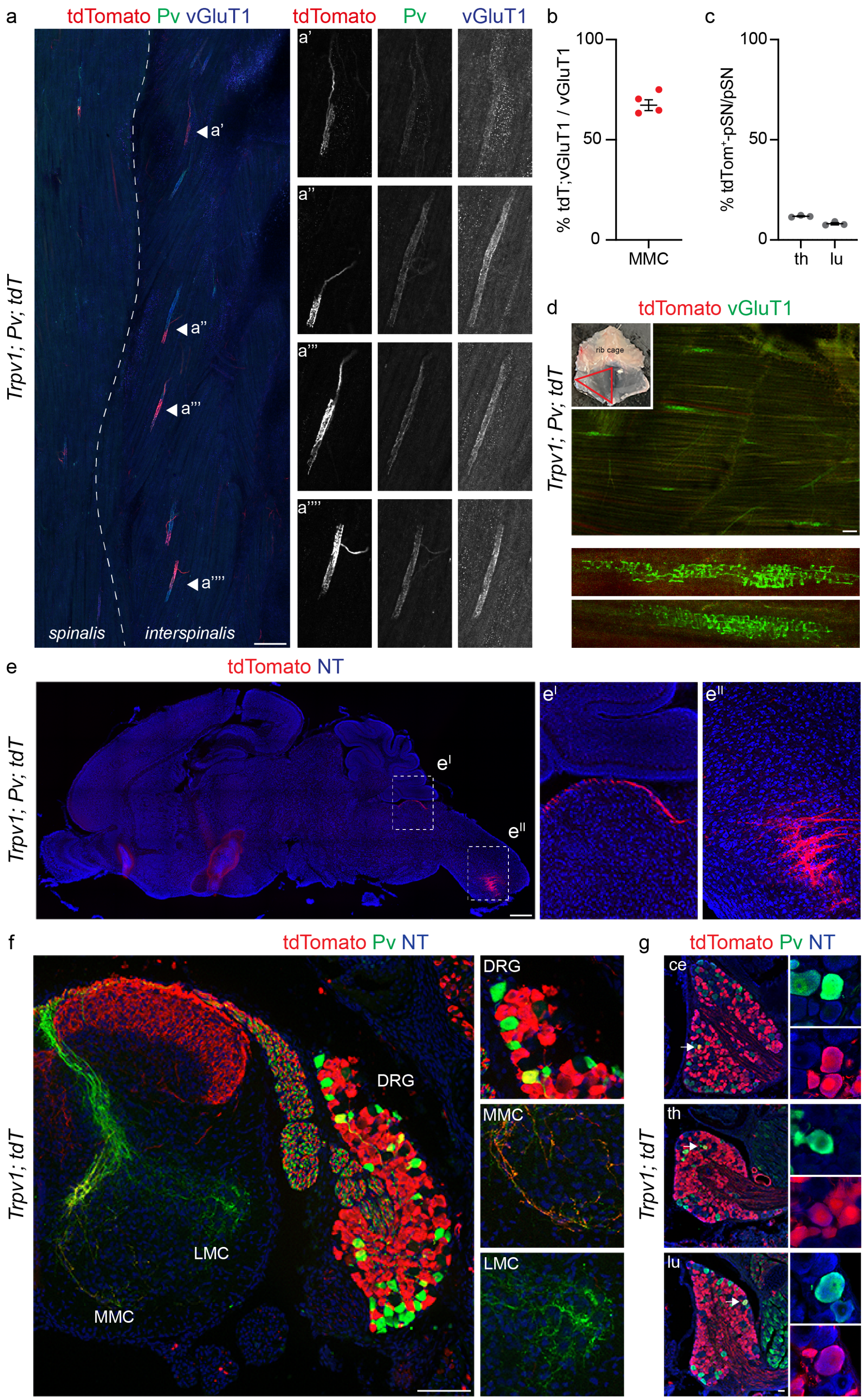

Genetic labeling of *Trpv1*$^+$ proprioceptors. a) Representative images of vGluT1$^+$; Pv$^+$; tdTomato$^+$ spindles in the back (spinalis and interspinalis) muscles of p7 *Trpv1*^{Cre}; *Pv*^{Flp}; *Ai65* mice. Scale bar: 100 µm. b) Percentage of vGluT1$^+$ boutons juxtaposed to ChAT$^+$ MMC neurons cell bodies and proximal dendrites labeled by tdTomato in p7 *Trpv1*^{Cre}; *Pv*^{Flp}; *Ai65* mice (mean ± SEM, n = 4 animals, 70 muscle spindles). c) Percentage of proprioceptors labeled in p7 *Trpv1*^{Cre}; *Pv*^{Flp}; *Ai65* mice at thoracic and lumbar levels (mean ± SEM, n = 3 animals). d) Representative image of vGluT1$^+$; tdTomato$^-$ spindles in the abdominal muscles of p7 *Trpv1*^{Cre}; *Pv*^{Flp}; *Ai65* mice. Scale bar: 100 µm. e) Representative sagittal brain section showing labeling of tdTomato$^+$ proprioceptive afferents in the brainstem (e^I) and cervical spinal cord (e^{II}) of p7 *Trpv1*^{Cre}; *Pv*^{Flp}; *Ai65* mice. Scale bar: 500 µm. f) Representative image of sensory neurons labeled in a p7 *Trpv1*^{Cre}; *Ai14* mice at lumbar level and high magnifications of DRG, MMC and LMC areas. Scale bar: 100 µm. g) Representative images of cervical (ce), thoracic (th), and lumbar (lu) DRG sections showing tdTomato$^+$; Pv$^+$ sensory neurons in p7 *Trpv1*^{Cre}; *Ai14* mice. Scale bar: 25 µm. Source data are provided as a Source Data file.

Supplementary Figure 3

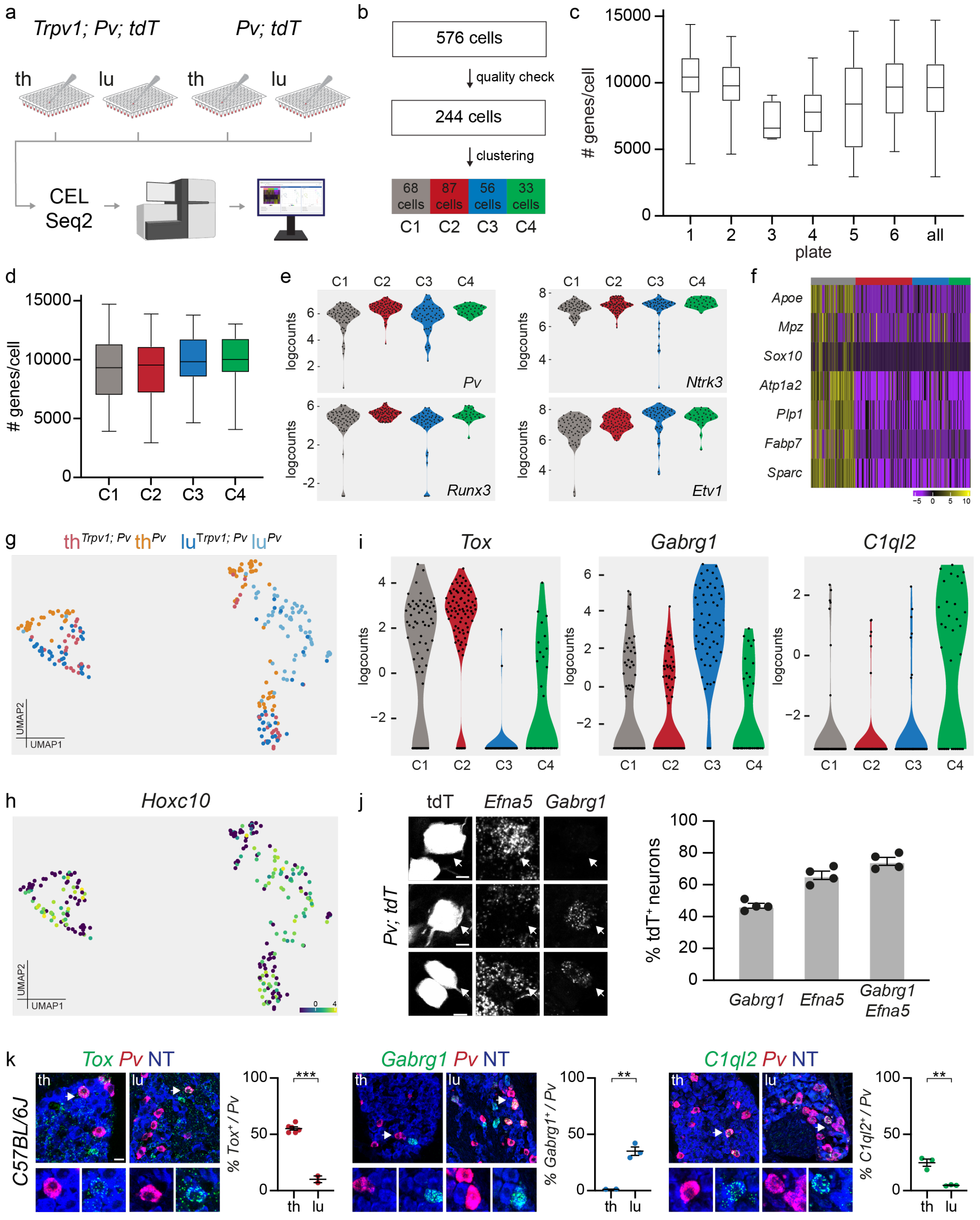

Transcriptome analysis at p1 and validation of proprioceptor muscle subtypes. a) Schematic representation of the single cell sorting strategy for neurons dissociated from p1 thoracic and lumbar DRG of *Trpv1*^{Cre}; *Pv*^{Flp}; *Ai65* and *Pv*^{Cre}; *Ai14* mice. b) Number of tdTomato^{+} DRG neurons sorted, analyzed after quality control and assigned to each cluster after bioinformatic analysis. c) Boxplots representing the number of genes per

cells found in each 96 well plate (#1 to #6) and on average (“all”). The boxes represent the 1st and 3rd quartiles (bounds of box), median (horizontal line), and whiskers extend to further than 1.5*IQR (interquartile range) from the hinges. d) Boxplots representing the number of genes per cells found in each cluster. The boxes represent the 1st and 3rd quartiles (bounds of box), median (horizontal line), and whiskers extend to further than 1.5*IQR (interquartile range) from the hinges. e) Violin plots showing expression (logcounts) of general proprioceptor markers (*Pv*, *Ntrk3*, *Runx3*, *Etv1*) at p1. f) Heatmap showing expression (logcounts) of glial cell markers in proprioceptor clusters at p1. g) UMAP visualization of clusters color coded to represent thoracic and lumbar origin of cells sorted from $Trpv1^{Cre}$; Pv^{Flp}; *Ai65* and Pv^{Cre}; *Ai14* mice. h) UMAP visualization of *Hoxc10* expression (logcounts) in proprioceptor clusters at p1. i) Violin plots showing expression (logcounts) of *Tox*, *Gabrg,* and *C1ql2* in proprioceptor clusters at p1. j) Representative smFISH images of *Gabrg1* (C3) and *Efna5* (C3) expression in $tdTomato^+$ lumbar DRG neurons of p1 Pv^{Cre}; *Ai14* mice (left) and quantification of percentage of $tdTomato^+$ sensory neurons expressing each marker alone or in combination (right) (mean ± SEM, n = 4 animals). k) Representative smFISH images and quantification of *Tox* (C2), *Gabrg1* (C3), and *C1ql2* (C4) expression in Pv^+ thoracic and lumbar DRG neurons of p1 wild type mice (mean ± SEM, n ≥ 2 animals, two-tailed t-test, ** $p < 0.01$, *** $p < 0.001$, p-values < 0.001 (*Tox*), 0.006 (*Gabrg1*), 0.003 (*C1ql2*). Scale bar: 25 µm. Source data are provided as a Source Data file.

Supplementary Figure 4

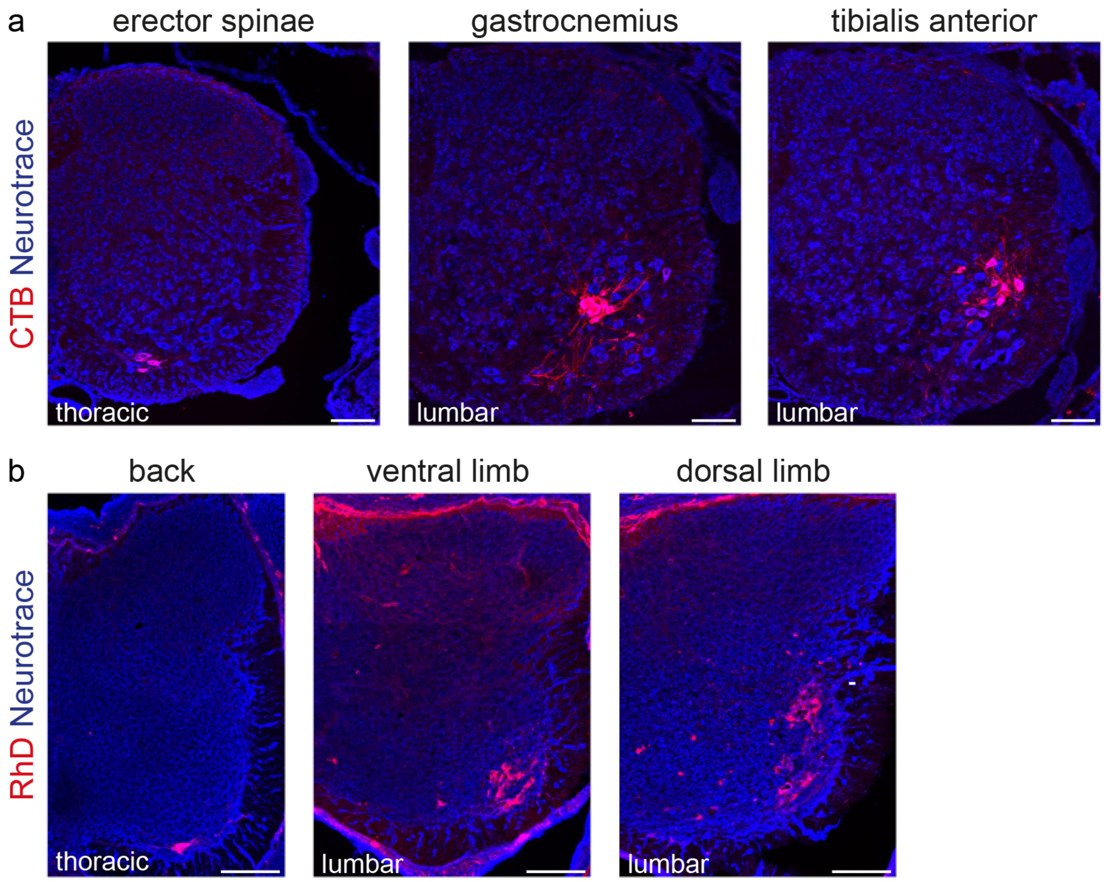

Retrograde labeling of epaxial and limb innervating motor neurons. a) Representative images of motor neurons retrogradely labeled after CTB injection in back (erector spinae) and hindlimb (gastrocnemius and tibialis anterior) muscles of wild-type mice. Scale bar:100 µm. b) Representative images of motor neurons retrogradely labeled after RhD injection in epaxial (back), ventral, and dorsal hindlimb muscles of e15.5 wild-type embryos. Scale bar: 100 µm.

Supplementary Figure 5

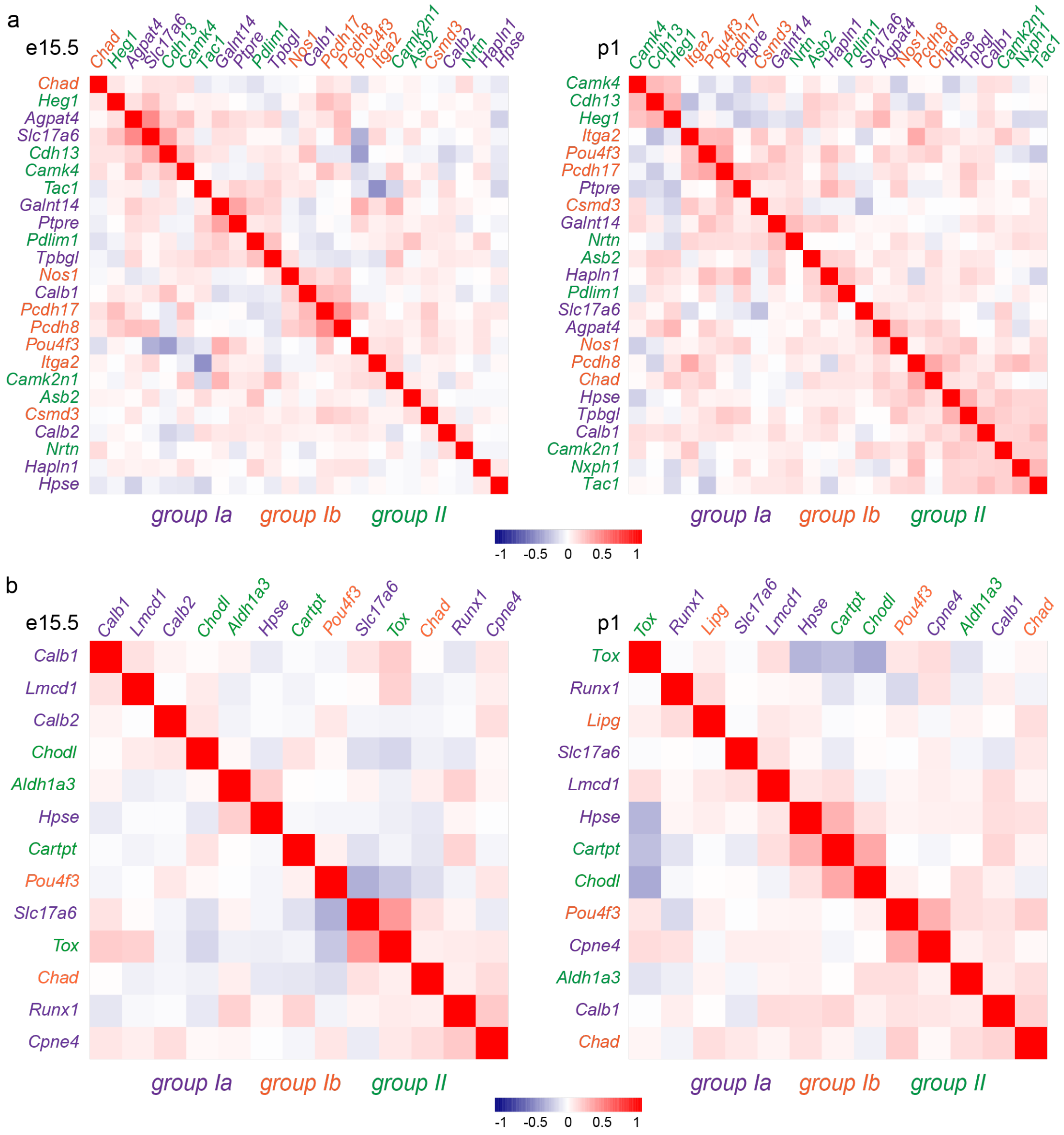

Signatures for "receptor-type" proprioceptors start emerging at p1. a) Heatmaps representing pairwise gene expression correlation values for group Ia (blue), group II (green), and group Ib (red) molecular signatures identified by Oliver and colleagues[1] at e15.5 (left) and p1 (right; Pearson's r using logcounts). b) Heatmaps representing pairwise gene expression correlation values for group Ia (blue), group II (green), and group Ib (red) molecular signatures identified by Wu and colleagues[2] at e15.5 (left) and p1 (right; Pearson's r using logcounts).

Supplementary Figure 6

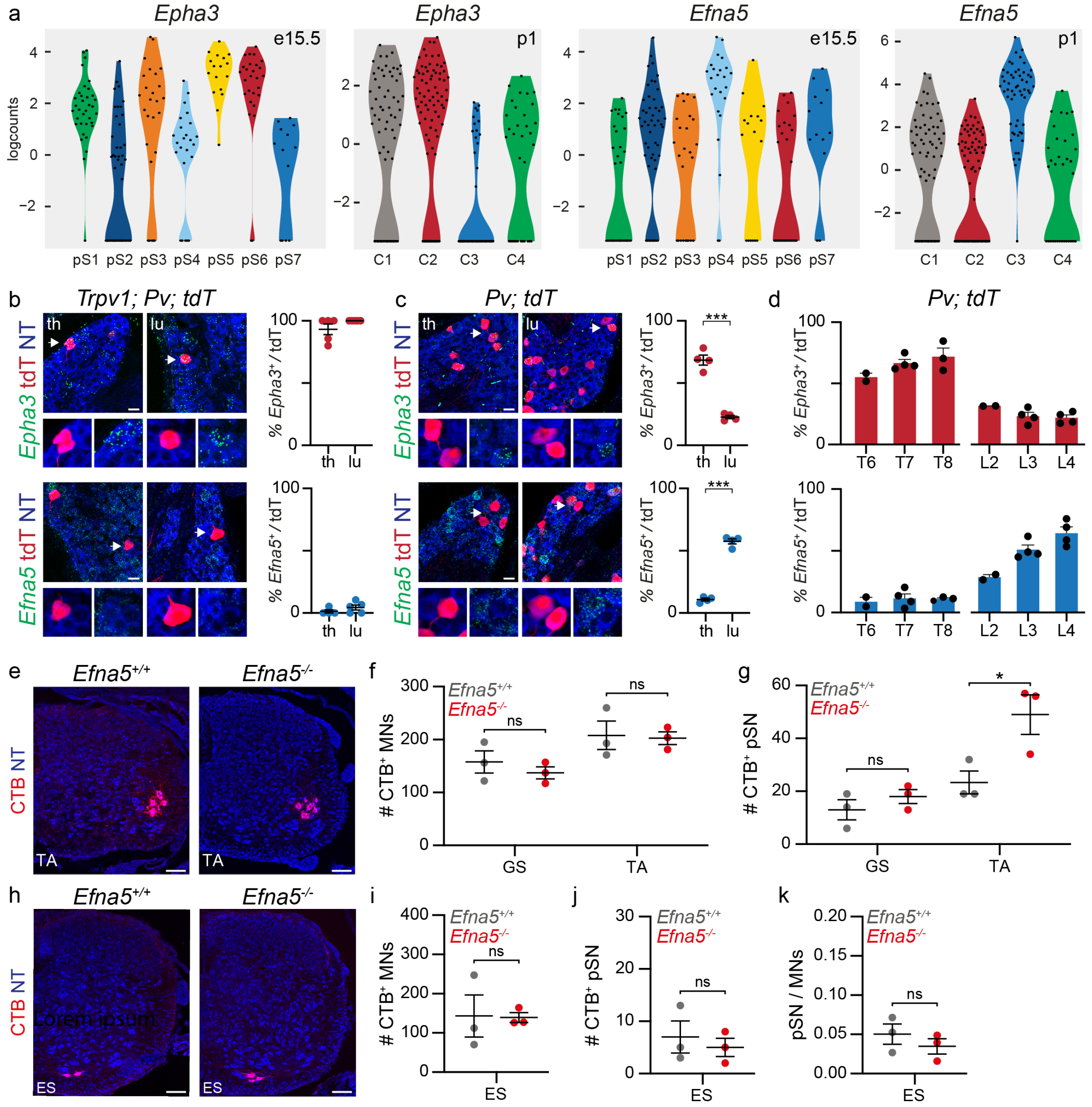

***Epha3* and *Efna5* expression defines proprioceptor muscle subtypes.** a) Violin plots of *Epha3* and *Efna5* expression (logcounts) at e15.5 and p1. b) smFISH images and quantification of *Epha3* (top) and *Efna5* (bottom) expression in tdTomato^{+} thoracic and lumbar DRG neurons of p1 *Trpv1^{Cre}; Pv^{Flp}; Ai65* mice (mean ± SEM, n ≥ 4 animals). Scale bar: 25 µm. c) smFISH images and quantification of *Epha3* (top) and *Efna5* (bottom) expression in tdTomato^{+} thoracic and lumbar DRG neurons of p1 *Pv^{Cre}; Ai14* mice (mean ± SEM, n = 4 animals, two-tailed t-test, *** $p < 0.001$). Scale bar: 25 µm. d) Distribution of *Epha3* and *Efna5* in tdTomato^{+} neurons from thoracic and lumbar

DRG of p1 *Pv^{Cre}; Ai14* mice (mean ± SEM, n ≥ 2 animals). e) Motor neurons labeled after CTB injection in the TA muscle of p1 *$Efna5^{+/+}$* and *$Efna5^{-/-}$* mice. Scale bar: 100 µm. f) Number of motor neurons labeled after CTB injection in the GS and TA muscles of p1 *$Efna5^{+/+}$* (gray) and *$Efna5^{-/-}$* (red) mice (mean ± SEM, n = 3 animals, two-tailed t-test, ns $p > 0.05$, p-values 0.44 GS and 0.86 TA). g) Number of proprioceptors (Pv^+) labeled after CTB injection in the GS and TA muscles of p1 *$Efna5^{+/+}$* (gray) and *$Efna5^{-/-}$* (red) mice (mean ± SEM, n = 3 animals, two-tailed t-test, ns $p > 0.05$, * $p < 0.05$, p-values 0.34 GS and 0.04 TA). h) Motor neurons retrogradely labeled after CTB injection in the ES muscle of p1 *$Efna5^{+/+}$* and *$Efna5^{-/-}$* mice. Scale bar: 100 µm. i) Number of motor neurons labeled after CTB injection in the ES muscle of p1 *$Efna5^{+/+}$* (gray) and *$Efna5^{-/-}$* (red) mice (mean ± SEM, n = 3 animals, two-tailed t-test, ns $p > 0.05$, p-value 0.95). j) Number of proprioceptors (Pv^+) labeled after CTB injection in the ES muscle of p1 *$Efna5^{+/+}$* (gray) and *$Efna5^{-/-}$* (red) mice (mean ± SEM, n = 3 animals, two-tailed t-test, ns $p > 0.05$, p-value 0.60). k) Ratio of proprioceptor (Pv^+) per motor neuron labeled after CTB injection in the ES muscle of p1 *$Efna5^{+/+}$* (gray) and *$Efna5^{-/-}$* (red) mice (mean ± SEM, n = 3 animals, two-tailed t-test, ns $p > 0.05$, p-value 0.39). Source data are provided as a Source Data file.

Supplementary Figure 7

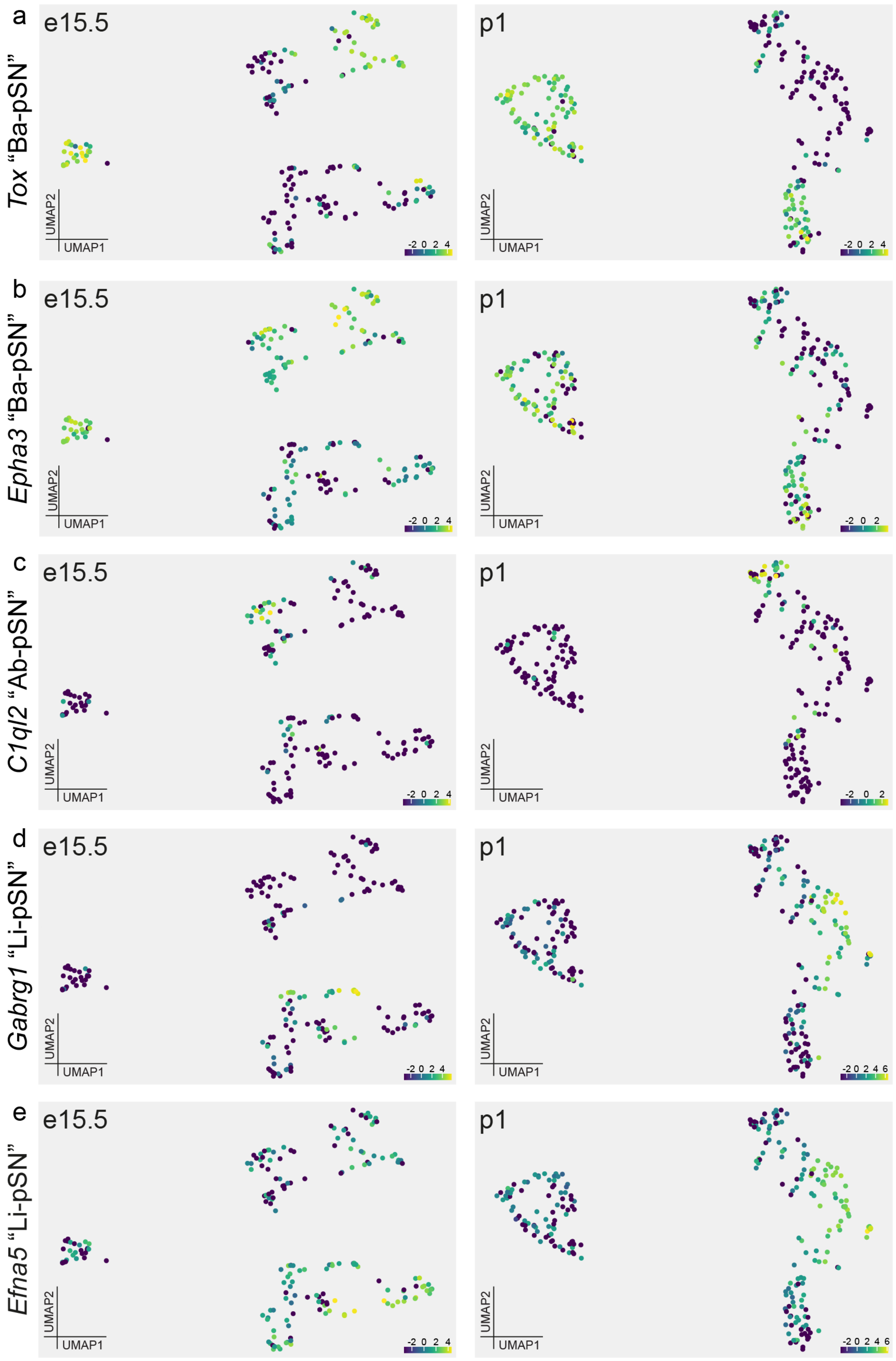

Expression of proprioceptor muscle-type markers in e15.5 and p1 clusters. UMAP visualizations of *Tox* (a), *Epha3* (b), *C1ql2* (c)*, Gabrg1* (d) and *Efna5*(e) expression (logcounts) at e15.5 and p1.

Supplementary References

1. Oliver, K. M. et al. Molecular correlates of muscle spindle and Golgi tendon organ afferents. Nat. Commun. **12**, 1451 (2021).
2. Wu, H. et al. Distinct subtypes of proprioceptive dorsal root ganglion neurons regulate adaptive proprioception in mice. Nat. Commun. **12**, 1026 (2021).
