# Supplementary material for: Molecular identity of proprioceptor subtypes innervating different muscle groups in mice
Source: Nat Commun. 2022 Nov 11;13:6867. doi: 10.1038/s41467-022-34589-8 (PMC9652284; doi:10.1038/s41467-022-34589-8)
Supplement: Supplementary file 3 — Description of Additional Supplementary Files [file 41467_2022_34589_MOESM3_ESM.pdf]

Description of Additional Supplementary Files

Supplementary Movie 1. Light-sheet imaging of a p7 lumbar spinal cord from a *Trpv1*^{Cre}*; Pv*^{Flp}*; Ai65* mouse.

Supplementary Movie 2. Light-sheet imaging of a p7 lumbar spinal cord from a *Pv*^{Cre}*; Ai14* mouse.
