# Supplementary material for: Molecular identity of proprioceptor subtypes innervating different muscle groups in mice
Source: Nat Commun. 2022 Nov 11;13:6867. doi: 10.1038/s41467-022-34589-8 (PMC9652284; doi:10.1038/s41467-022-34589-8)
Supplement: Supplementary file 6 — Reporting Summary [file 41467_2022_34589_MOESM6_ESM.pdf]

nature portfolio

Corresponding author(s): Niccolo Zampieri

Last updated by author(s): Oct 24, 2022

Reporting Summary

Nature Portfolio wishes to improve the reproducibility of the work that we publish. This form provides structure for consistency and transparency in reporting. For further information on Nature Portfolio policies, see our Editorial Policies and the Editorial Policy Checklist.

Statistics

For all statistical analyses, confirm that the following items are present in the figure legend, table legend, main text, or Methods section.

n/a	Confirmed	
☐	☒	The exact sample size (n) for each experimental group/condition, given as a discrete number and unit of measurement
☐	☒	A statement on whether measurements were taken from distinct samples or whether the same sample was measured repeatedly
☐	☒	The statistical test(s) used AND whether they are one- or two-sided<br>*Only common tests should be described solely by name; describe more complex techniques in the Methods section.*
☒	☐	A description of all covariates tested
☒	☐	A description of any assumptions or corrections, such as tests of normality and adjustment for multiple comparisons
☐	☒	A full description of the statistical parameters including central tendency (e.g. means) or other basic estimates (e.g. regression coefficient) AND variation (e.g. standard deviation) or associated estimates of uncertainty (e.g. confidence intervals)
☐	☒	For null hypothesis testing, the test statistic (e.g. F, t, r) with confidence intervals, effect sizes, degrees of freedom and P value noted<br>*Give P values as exact values whenever suitable.*
☒	☐	For Bayesian analysis, information on the choice of priors and Markov chain Monte Carlo settings
☒	☐	For hierarchical and complex designs, identification of the appropriate level for tests and full reporting of outcomes
☐	☒	Estimates of effect sizes (e.g. Cohen's d, Pearson's r), indicating how they were calculated

Our web collection on statistics for biologists contains articles on many of the points above.

Software and code

Policy information about availability of computer code

Data collection	Confocal images were acquired with a LSM 800 microscope from Zeiss using ZENblue v 3.4 8 (Zeiss), Lightsheet images were acquired with a Zeiss Lightsheet microscope Z.1 using the ZENblack v 3.1 (Zeiss) software.
Data analysis	Arivis Vision4D v 3.5.1 Arivis AG, Imaris v 9.8.0 Oxford Instruments, Prism - GraphPad v 9.3.1 GraphPad Software, Adobe Photoshop v 25.4.1 Adobe (count tool), ImageJ2 v 2.3.0/153f NIH, R v3.6.2, scruff v1.4.0 package (R package version 1.12.0), Scater v1.14.6 R package, scran v1.14.1 buildKNNGraph and cluster_walktrap

For manuscripts utilizing custom algorithms or software that are central to the research but not yet described in published literature, software must be made available to editors and reviewers. We strongly encourage code deposition in a community repository (e.g. GitHub). See the Nature Portfolio guidelines for submitting code & software for further information.

Data

Policy information about availability of data

All manuscripts must include a data availability statement. This statement should provide the following information, where applicable:

- Accession codes, unique identifiers, or web links for publicly available datasets
- A description of any restrictions on data availability
- For clinical datasets or third party data, please ensure that the statement adheres to our policy

All data is available in the main text and supplemental materials. Sequencing data is available through the NCBI GEO database (accession code: GSE190605), https://www.ncbi.nlm.nih.gov/geo/query/acc.cgi?acc=GSE190605. Source data are provided with this paper.

Field-specific reporting

Please select the one below that is the best fit for your research. If you are not sure, read the appropriate sections before making your selection.

- [x] Life sciences
- [ ] Behavioural & social sciences
- [ ] Ecological, evolutionary & environmental sciences

For a reference copy of the document with all sections, see nature.com/documents/nr-reporting-summary-flat.pdf

Life sciences study design

All studies must disclose on these points even when the disclosure is negative.

Sample size	Based on our hypothesis to identify at least three distinct clusters of proprioceptors at embryonic stage e15.5 and considering that our genetic approach might also label low-threshold mechanoreceptors we aimed to analyse 480 thoracic cells and 480 lumbar cells. We calculated the number of cells using the online application of the Satija lab (https://satijalab.org/howmanycells/) considering the assumed number of cell types, the minimum fraction of the rarest cell type, and the minimum number of desired cells per type. For each experiment cells of Pv-tdt; e15.5 embryos (at least n = 4) from 1 litter were pooled and sorted into 96-well plates, 5 plates with cells from thoracic DRGs and 5 plates with cells from lumbar DRGs were collected. Embryos of n=6 litters were used in total.<br>The same web application from the Satija lab was used to estimate the cell number for the p1 single-cell-transcriptome analysis, also considering that our intersectional approach labels a specific proprioceptor muscle-type subset and that the total number of labelled neurons is low. The following number of cells from the different mouse lines were collected for p1 scRNAseq experiments: Trpv1Cre-Basbaum; PvFlp; Ai65; p1; 96 thoracic and 96 lumbar; cells; Trpv1Cre-Hoon; PvFlp; Ai65; p1; 96 thoracic and 96 lumbar cells; PvCre; Ai14; p1; 96 thoracic and 96 lumbar cells. For multiplexed FISH, immunohistochemistry, and retrograde tracing experiments we used at least n=3 animals.
Data exclusions	For scRNAseq experiments we individually removed low-quality cells based on low total gene counts (> quantile 0.3), low gene abundance (> quantile 0.3), and high mitochondrial gene values cells (< quantile 0.75).
Replication	For each scRNAseq experiment, we sequenced single cells from 10 96-well plates (e15.5) and 6 96-well plates) (p1) containing cells from at least n=6 animals of at least 3 different litters. For validation of scRNAseq data using multiplexed FISH as well as for immunohistochemistry, and retrograde tracing experiments at least n=3 biological replicates were used.
Randomization	The scRNAseq protocol relies on sorting cells from a cell suspension, which already leads to a random selection of cells from the labelled population. For multiplexed FISH, immunohistochemistry, and retrograde tracing experiments no randomization was performed as no experimental groups were used.
Blinding	For scRNAseq experiments and validation of scRNAseq data (multiplexed FISH, immunohistochemistry, and retrograde tracing experiments) blinding was not necessary because we did not compare experimental and control groups. For experiments involving Efna5 mutant mice standard blinding procedures were used.

Reporting for specific materials, systems and methods

We require information from authors about some types of materials, experimental systems and methods used in many studies. Here, indicate whether each material, system or method listed is relevant to your study. If you are not sure if a list item applies to your research, read the appropriate section before selecting a response.

Materials & experimental systems

n/a	Involved in the study
[ ]	[x] Antibodies
[x]	[ ] Eukaryotic cell lines
[x]	[ ] Palaeontology and archaeology
[ ]	[x] Animals and other organisms
[x]	[ ] Human research participants
[x]	[ ] Clinical data
[x]	[ ] Dual use research of concern

Methods

n/a	Involved in the study
[x]	[ ] ChIP-seq
[x]	[ ] Flow cytometry
[x]	[ ] MRI-based neuroimaging

Antibodies

Antibodies used	Primary antibodies:<br>anti-ChAT (Goat); Millipore (#AB144P); 1:200<br>anti-Pv (Chicken); Jessell lab (#CU1664); 1:8000; de Nooij et al., 2013<br>anti-vGluT1 (Guinea-Pig); Jessell lab (#CU1328); 1:5000; de Nooij et al., 2013<br>anti-dsRed Rabbit; Takara (#632496); 1:1000<br>anti-RFP (Rabbit); Rockland (#600-401-379); 1:500<br><br>Secondary antibodies

	Alexa Fluor® 488 AffiniPure Donkey Anti-Goat IgG (H+L); Jackson Immuno Research Laboratories (#705-545-003); 1:1000<br>Cy™5 AffiniPure Donkey Anti-Goat IgG (H+L); Jackson Immuno Research Laboratories (#705-175-147); 1:250<br>Fluorescein (FITC) AffiniPure Donkey Anti-Chicken IgY (IgG) (H+L); Jackson Immuno Research Laboratories (#703-095-155); 1:1000<br>Alexa Fluor® 488 AffiniPure Donkey Anti-Guinea Pig IgG (H+L); Jackson Immuno Research Laboratories (#706-545-148); 1:1000<br>Cy™3 AffiniPure Donkey Anti-Rabbit IgG (H+L); Jackson Immuno Research Laboratories (#711-165-152); 1:1000
Validation	Primary antibodies were validated by examining the known tissue-specific expression patterns based on prior publications and comparing them with in-situ hybridization data/experiments. Further information can be found on suppliers' websites and/or references listed in the primary antibody list. Validation for secondary antibodies is available on manufacturers' websites.

Animals and other organisms

Policy information about studies involving animals; ARRIVE guidelines recommended for reporting animal research

Laboratory animals	Trpv1Cre-Basbaum : B6.129-Trpv1tm1(cre)Bbm/J The Jackson Laboratory Stock No: 017769, early postnatal animals were used for experiments.<br>Trpv1Cre-Hoon : Tg(Trpv1-cre)1Hoon Hoon Lab MGI:4942415, early postnatal animals were used for experiments.<br>PvFlp:B6.Cg-Pvalbtm4.1(flpo)Hze/J The Jackson Laboratory Stock No: 022730, early postnatal animals were used for experiments.<br>PvCre:Pvalbtm1(cre)Arbr The Jackson Laboratory Stock No: 017320, early postnatal animals were used for experiments.<br>PvtdTom:C57BL/6-Tg(Pvalb-tdTomato)15Gfng/J The Jackson Laboratory Stock No: 027395, embryonic animals (e15.5) of unknown sexes were used for experiments.<br>Ai14:B6;129S6-Gt(ROSA)26Sortm14(CAG-tdTomato)Hze/J The Jackson Laboratory Stock No: 007908, early postnatal animals were used for experiments.<br>Ai65:B6;129S-Gt(ROSA)26Sortm65.1(CAG-tdTomato)Hze/J The Jackson Laboratory Stock No: 021875, early postnatal animals were used for experiments.<br>Efna5 :Efna5tm1Ddmo/J The Jackson Laboratory Stock No: 005992, early postnatal animals were used for experiments.<br>If not indicated otherwise animals of both sexes were used in all experiments.
Wild animals	No wild animals were used in this study
Field-collected samples	No field-collected samples were used in this study.
Ethics oversight	All experiments were performed in compliance with the German Animal Welfare Act and approved by the Regional Office for Health and Social Affairs Berlin (LAGeSo) under license numbers G0148/17 and G0191/18.

Note that full information on the approval of the study protocol must also be provided in the manuscript.
